# Innate Lymphoid Cells: A Link between the Nervous System and Microbiota in Intestinal Networks

**DOI:** 10.1155/2019/1978094

**Published:** 2019-01-20

**Authors:** Lin Han, Xin-miao Wang, Sha Di, Ze-zheng Gao, Qing-wei Li, Hao-ran Wu, Qing Wang, Lin-hua Zhao, Xiao-lin Tong

**Affiliations:** ^1^Department of Endocrinology, Guang'anmen Hospital of China, China Academy of Chinese Medical Sciences, Beijing 100054, China; ^2^Laboratory of Molecular and Biology, Guang'anmen Hospital of China, China Academy of Chinese Medical Sciences, Beijing 100053, China; ^3^Beijing University of Chinese Medicine, Beijing 100029, China

## Abstract

Innate lymphoid cells (ILCs) are a novel family of innate immune cells that act as key coordinators of intestinal mucosal surface immune defense and are essential for maintaining intestinal homeostasis and barrier integrity by responding to locally produced effector cytokines or direct recognition of exogenous or endogenous danger patterns. ILCs are also involved in the pathogenesis of inflammatory bowel disease (IBD). Many studies have demonstrated the occurrence of crosstalk between ILCs and intestinal microbiota, and ILCs have recently been shown to be connected to the enteric nervous system (ENS). Thus, ILCs may act as a key link between the nervous system and microbiota in intestinal networks. In this review, we briefly summarize the role of the ILCs in the intestinal tract (particularly in the context of IBD) and discuss the relationship between ILCs and the microbiota/ENS.

## 1. Introduction

Innate lymphoid cells (ILCs) play an important role in the immune regulatory network, which are not only the effector cells of innate immunity but also mediate acquired immunity-related functions. Recent studies have identified a special subset of lymphocytes in the human and mouse mucosal systems (e.g., the intestines and lungs) and other critical organs (e.g., the liver) that are related to regional immunization. These lymphocytes are derived from a common lymphoid progenitor (CLP), such as T cells and B cells, and depend on the master lymphocyte cytokine receptor interleukin- (IL-) 2 receptor common *γ* chain and the expression of inhibitor of DNA binding 2 (Id2) [1, 2]. These cells can be distinguished from adaptive lymphocytes by the absence of functionally rearranged antigen-specific receptors that recognize “nonself” structures and participate in the innate immune response. Therefore, these cells have been designated ILCs, including classic cytotoxic natural killer (NK) cells and lymphoid tissue inducer (LTi) cells.

Most CLPs are developed in the bone marrow, whereas mature ILCs are mainly enriched in peripheral tissues such as the gastrointestinal tract, lung, liver, and skin. There are exceptions, for instance, LTi cells are from the fetal liver to the periphery [3]. Recent studies from parabiosis experiments have confirmed that the vast majority of ILCs are tissue-resident [4]. Mature ILCs have been further categorized into three groups based on differences in effector cytokine production [5–7]. Group 1 ILCs (ILC1s), including NK cells, have the capacity to produce the T helper- (Th-) 1 cell signature cytokines interferon-*γ* (IFN-*γ*) and tumor necrosis factor-*α* (TNF-*α*), and their function is regulated by the transcription factor T-bet or eomesodermin (Eomes) following stimulation with the proinflammatory cytokines IL-12, IL-15, and IL-18 [8–10]. Group 2 ILCs (ILC2s) express and require the transcription factors Gata binding protein 3 (GATA-3) and/or retinoic acid receptor- (RAR-) related orphan receptor-*α* (ROR*α*) and are capable of secreting Th2 cell-type cytokines, such as IL-4, IL-5, IL-9, and IL-13 [11, 12], in response to IL-25, IL-33, or thymic stromal lymphopoietin [13]. Lastly, group 3 ILCs (ILC3s), including LTi cells, are defined by the expression of the transcription factor ROR*γ*t and have been shown to be associated with the production of IL-17A, IL-17F, IL-22, and colony stimulating factor 2 (CSF2, also known as granulocyte-macrophage CSF) in response to stimulation with IL-23 and IL-1*β* [13], which are characteristic of Th17/Th22 cells [5, 7, 14]. Different ILC subtypes have different functions, and maintenance of the steady state of the body depends on the coordination of and interactions among the three subtypes.

ILCs are unique in the innate immune system in that they may produce and secrete cytokines that are classically regarded as CD4^+^ Th cell products and are considered to be the “mirror image” of CD4^+^ Th cells. Compared with adaptive lymphocytes, ILCs are relatively rare in lymphoid tissue and are mainly deposited on barrier surfaces, such as the skin, intestine, lungs, fat, and mucosa-associated lymphoid tissue. Among these tissues, the intestine is a secondary lymphoid organ and the largest immune system-related organ in the body. Notably, the most common intestinal disease is inflammatory bowel disease (IBD), which encompasses Crohn's disease (CD) and ulcerative colitis (UC). IBD is a chronic, recurrent inflammatory disease of the intestine and strongly impairs the quality of life of patients [13]. Moreover, some studies have shown that IBD may increase the risk of cancer [15, 16]. Currently, the molecular mechanisms mediating IBD remain unclear, and clinical treatments are not satisfactory. However, studies have shown that the pathogenesis of this disease involves genetic, immunologic, infectious, environmental, and mental factors. Additionally, IBD is known to involve impairment of the integrity of the mucosal epithelial barrier. Because ILCs play an important role in mucosal homeostasis and are involved in a critical feedback loop in which damaged epithelium activates ILCs to restore epithelial barrier function [17–19], ILCs may participate in the pathogenesis of IBD.

Many researchers have attempted to elucidate the relationships between ILCs and intestinal microbes, and recent studies have shown that there is a link between ILCs and the enteric nervous system (ENS) [20–22]; however, the details of this connection are still unclear. In this review, we discuss the roles of ILCs in the intestine, particularly in the context of IBD, and trace the connections among ILCs, the intestinal microbiota, and the ENS in intestinal networks.

## 2. Overview of ILCs in the Intestinal Tract

**Figure 1:**
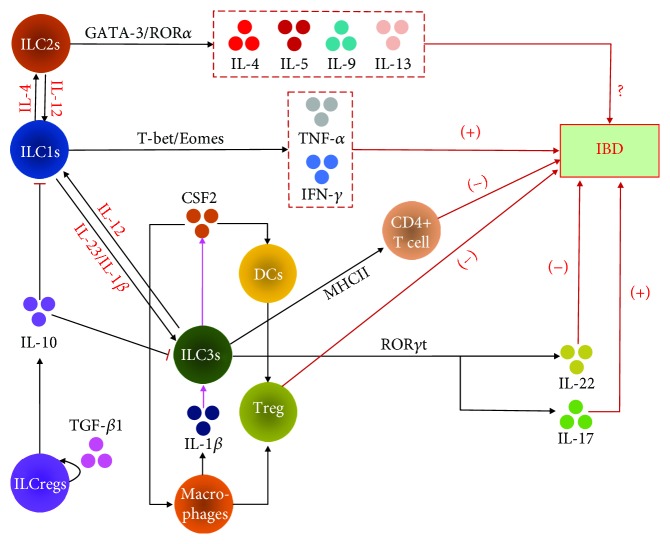
The main mechanisms regulating ILCs in IBD. The schematic shows the three ILC subgroup transcription factors and their secretory cytokines, which play proinflammatory (+) or anti- inflammatory (-) roles in IBD, and inhibiting “arrow” means suppression effects on ILCs. Particularly for ILC3s, macrophages secrete IL-1*β*, which induces ROR*γ*t^+^ ILC3s to produce colony stimulating factor 2 (CSF2) (shown by purple arrow). CSF2 then acts on dendritic cells (DCs) and macrophages to promote the secretion of regulatory factors and induce the transformation of immature T cells into mature regulatory T cells (Tregs), which are essential for inhibiting inflammation and maintaining intestinal homeostasis. Natural cytotoxicity receptor^−^ (NCR^−^) ILC3s express high levels of major histocompatibility complex (MHC) II, which is involved in processing and presenting antigens and can limit the response between CD4^+^ T cells and intestinal commensal bacteria, thereby inhibiting inflammation mediated by CD4^+^ T cells to prevent IBD. ILCregs can protect against innate intestinal inflammation by secreting IL-10 to suppress the activation of ILC1s, ILC3s, and autocrine TGF-*β*1 for the expansion and survival of ILCregs during intestinal inflammation. Furthermore, ILCs could mutually transform via induction by specific cytokines (shown by red font).

ILCs are important populations of innate immune effectors and are mainly distributed in the intestinal mucosa, including the intraepithelial compartment and lamina propria. ILC1s are the most abundant ILC population in the intraepithelial compartment in both the small and large intestines, whereas ILC2 and ILC3 are the dominant populations in the lamina propria in the large and small intestines, respectively [23]. Moreover, a study considered that ILCs, which are educated in mesenteric lymph node (MLN) and then home to the intestine, have sophisticated migration programs that undergo retinoic acid- (RA-) dependent homing receptor switching in a shared, yet subset-specific manner. However, the model they used and the data they tested did not directly prove this migration, which needs more evidences [12]. Additionally, another study showed that ILCs in the gut can be locally renewed and expanded in response to acute environmental challenges, such as helminth infection [4]. Although the ILC population in the intestine is only a fraction of the total lymphocytes, ILCs can promote lymphoid tissue genesis, intestinal mucosal barrier protection, gut microbiota and anti-infection immune regulation, tissue repair coordination, and tissue reconstruction, mainly via secretion of effector cytokines and interactions with Leydig cells or other immune cells [5, 24]. Furthermore, ILCs have a certain degree of plasticity, some subgroups will transform into each other when their surrounding microenvironment changes or during the development of certain diseases. The main regulatory mechanisms of ILCs in IBD are shown in Figure 1.

### 2.1. Group 1 ILCs

Group 1 ILCs include cytotoxic conventional (c) NK cells, which were first discovered in 1975 [25], and noncytotoxic ILC1 family. All ILC1s produce IFN-*γ* and TNF-*α*, and the physiological roles of ILC1s involve immune responses to intracellular pathogens [26] and tumors [13, 27]. cNK cells are effector cells present in the lamina propria and have roles in antitumor responses via secretion of perforin to kill target cells. In addition to direct killing of target cells, cNK cells can produce the proinflammatory factor IFN-*γ* during early inflammation [28]. Some other proinflammatory cytokines, such as IL-15 and IL-21, can induce and activate NK cells to secrete abundant amounts of IFN-*γ* and TNF-*α* [29]. In comparison with healthy controls, the number of CD16^+^ NK cells present in the lamina propria of patients with CD and UC is substantially increased. However, CD161^+^ NK cells in the colonic lamina propria have also been shown to have anti-inflammatory roles, and the number of CD161^+^ NK cells in patients with UC is obviously decreased [30]. Therefore, further studies are needed to elucidate the regulatory mechanisms mediating these processes.

TNF-*α* and IFN-*γ* are both characteristic proinflammatory cytokines. TNF-*α* plays a key role in early rapid immunity via a pathway independent of MHC molecules and antibodies. IFN-*γ* is closely linked to IBD [31]. The number of intraepithelial ILC1s is increased in patients with CD [32] and in mice with anti-CD40-induced colitis [33], thereby contributing to intestinal inflammation via secretion of IFN-*γ* [29, 34] and forming a pathological environment with a high concentration IFN, and this induced inflammation can be ameliorated by depletion of ILC1s [35].

### 2.2. Group 2 ILCs

In the intestinal tract, ILC2s are more homogeneous cells than ILC1s and ILC3s. Group 2 ILCs function to express transcription factor GATA3 and produce type 2 cytokines such as IL-4, IL-5, IL-9, and IL-13. ILC2s are important in the clearance of helminth [36] and viral infections [37] and in the progression of asthma and lung allergies [38, 39]. Studies have indicated that crosstalk occurs between ILC2s and CD4^+^ Th2 cells during infection. This crosstalk is important for optimizing antihelminthic responses through a transition from innate to adaptive immunological pathways [40], and depletion of ILC2s in mice disrupts Th2 responses [41]. However, in the absence of ILC2s, a normal Th2 response may still occur [42], suggesting that ILC2-derived IL-13 and the underlying antigen-presenting functions of ILC2s may not be essential for enhancing Th2 responses.

The role of ILC2s in IBD is still unclear. The proportion of IL-13-producing ILC2s is increased in the intestinal mucosa of patients with CD, and these cells also produce IFN-*γ* [43]. A study showed that the number of IL-13-producing ILC2s is increased in an oxazolone-induced UC mouse model [44]. Additionally, production of the type 2 cytokines IL-4, IL-5, IL-9, and IL-13 is related to the severity of IBD [45–47], and neutralization of the central cytokines IL-4 and IL-13 has been shown to control experimental intestinal inflammation owing to the involvement of type 2 responses in proinflammatory pathways at the enteric mucosa. Nevertheless, neutralization of human IL-13 had no therapeutic effect in patients with UC. Thus, although ILC2s may be involved in the pathogenesis of IBD, additional studies are needed to elucidate the specific mechanisms.

### 2.3. Group 3 ILCs

Group 3 ILCs include LTi cells, which were first discovered in 1992 [48], and postnatal ILC3 cells. Compared with the other ILCs, ILC3s are the predominant population in the ileum and colon [49]. LTi cells are involved in the development of lymphoid organs during embryogenesis by modulating the secretion of lymphotoxin-*β* and TNF-*α*. In addition, LTi cells can express IL-17A and IL-22 [13]. Additionally, ILC3s can be subdivided into two subsets according to the expression of natural cytotoxicity receptor (NCR) [5]. NCR^+^ ILC3 cells express NK markers (NKp46 in mice and NKp44 in humans) and secrete IL-22 but little IL-17, whereas NCR^−^ ILC3 cells produce IL-17 but limited amounts of IL-22 [5, 7]. Notably, the healthy human intestine contains primarily NCR^+^ ILC3 [50]. Compared with other ILC subtypes, ILC3s are particularly relevant to the intestinal tract. ILC3s, particularly LTi cells, contribute to tertiary lymphoid organogenesis [51–53], the containment of commensal bacteria [54], and the clearance of bacterial infections in the gut [55, 56]. Additionally, ILC3s are major regulators of the pathology of IBD [57, 58].

There is a relative decrease in the number of IL-22^+^ ILC3s in the intestinal mucosa of IBD animal models or patients with IBD. IL-22 has protective effects on intestinal mucous membranes. NCR^+^ ILC3s secrete IL-22 through interactions with aromatic hydrocarbon receptor (AHR) from gut microbes and food, and IL-22 then acts on nonhematopoietic cells (such as epithelial cells) through interactions with heterodimeric receptors and the signal transducer and activator of transcription 3 (STAT3) pathway, mediating mucosal wound healing responses and promoting the proliferation and fucosylation of epithelial cells to maintain the integrity of the intestinal barrier [13, 59]. IL-22 deficiency causes intestinal mucosal barrier damage, leading to exposure of intestinal tissue to a large number of antigens. Abnormal immune responses are then induced in the host, leading to the development of IBD. Excessive production of IL-22 leads to pathological abnormalities such as tumor growth in a mouse model of colorectal cancer [60].

Inflammatory CD4^+^ T cell responses to commensal bacteria are related to the pathogenesis of IBD. NCR^−^ ILC3s express high levels of MHC class II gene (MHCII) molecules, which are involved in processing and presenting antigens, thereby limiting the response between CD4^+^ T cells and intestinal commensal bacteria and inhibiting inflammation mediated by CD4^+^ T cells [61, 62]. Furthermore, MHCII levels are reduced in pediatric patients with IBD [62]. ROR*γ*t^+^ ILCs are the primary source of CSF2 in the intestine. IL-1*β*, which is secreted by macrophages after identifying pathogenic bacteria or symbiotic bacteria, stimulates CSF2 production by ILC3 in the intestinal mucosa. This could in turn act on dendritic cells and macrophages to promote the secretion of regulatory factors, such as RA and IL-10, which induce the transformation of immature T cells into mature regulatory T cells (Tregs), these cells are essential for inhibiting inflammation and maintaining intestinal homeostasis [63]. Recent studies have also shown that IL-10 removes damaged mitochondria by blocking the metabolism of macrophages and promoting mitophagy, thereby suppressing inflammation [64].

Cytokines secreted by ILC3s have different effects in IBD. In addition to playing important roles in limiting chronic intestinal inflammation and maintaining tissue homeostasis, some ILC3s (IL-17-producing NCR^−^ ILC3 cells) also have proinflammatory effects in IBD. Researchers have shown that the number of IL-17-producing ILC3s is significantly increased in inflamed intestines in patients with CD but not in patients with UC [58]. NCR^−^ ILC3 cells mainly secrete IL-17. Recent evidence has strongly suggested that IL-17-producing ILC3s drive colonic inflammation during *Helicobacter hepaticus* infection, and the number of NCR^−^ ILC3s was significantly increased in the intestinal tract of colitis model mice, resulting in activation of mononuclear macrophages via secretion of IL-17 and other cytokines and then inducing a series of mucosal inflammatory responses [65, 66]. Furthermore, IFN-*γ* secreted by ILC3s also has proinflammatory effects, as described below. Excessive production of IL-17A and IFN-*γ* would destroy the intestinal barrier and induce IBD.

### 2.4. Other ILCs

In addition to the three ILC subsets, new cells have been found to be closely associated with ILCs, however, the definitions of these cells remain unclear. Numerous studies have shown that ILCs exhibit plasticity and that plastic changes among ILCs are likely to cause skewing of the functionally plastic ILC subsets due to the microenvironment, which will also be stated in this part.

iCD8*α* cells are innate lymphocytes in the intestinal epithelium. These cells are derived from the lymphatic system and mediate innate immunity; thus, they are easily confused with ILCs. However, the development of iCD8*α* cells does not require the transcriptional suppressor Id2, and these cells therefore cannot be classified as ILCs [67], but are instead thought to exist at the edge of the intestinal immune system [68]. Moreover, these cells also participate in the pathogenesis of IBD. In a mouse model of colitis induced by anti-CD40 antibodies, iCD8*α* cells secrete abundant granzymes to enhance intestinal inflammation, which may promote infiltration of molecules into the intestinal epithelium [69].

ILCregs, a regulatory subpopulation of ILCs, have been identified in mouse and human intestines and have been shown to be regulated by the translational regulator Id3 [70]. ILCregs have been recently found to contribute protection against innate intestinal inflammation by secreting IL-10 to suppress the activation of ILC1s and ILC3s. In addition, autocrine transforming growth factor- (TGF-) *β*1 produced by ILCregs is required for the expansion and survival of ILCregs during intestinal inflammation [70].

Some ILCs are plastic cells that can adopt another ILCs fate depending on environmental cues. Following stimulation with IL-12, some ILC3s have the ability to upregulate T-bet and downregulate ROR*γ*t both in vitro and in vivo [55, 57, 71]. These changes promote the secretion of IFN-*γ* to activate macrophages and other mononuclear phagocytes and induce the development of IBD [72]. IL-7 has also been shown to maintain stable expression of ROR*γ*t and inhibit the transformation of ILC3s into ILC1-like cells, thereby reducing the inflammatory response [73]. The conversion from ILC3 to ILC1 was accompanied via downregulation of AHR [74]. Furthermore, researchers found that differentiation of ILC3s into ILC1s might be reversible. The transformation of CD127^+^ ILC1s into ILC3s is induced by IL-23 and IL-1*β*. This process is dependent on the transcription factor ROR*γ*t and is enhanced by retinoic acid [75]. Accordingly, the subtle balance between ILC1s and ILC3s ensures tissue integrity and maintains intestinal immune defense ability. In addition, plasticity has also been observed in ILC2s, which are modulated by IL-12 and express the Th1 cytokine IFN-*γ* under inflammatory conditions [43], in turn, IL-4 could convert ILC1s into ILC2s [76].

## 3. Crosstalk between ILCs and Gut Microbiota

Many studies have examined the roles of ILCs in the intestinal tract with a focus on their relationships with the intestinal microbiota. The gut microbiota is an essential component of the intestine, and its highly dynamic balance is vital for maintaining intestinal health and preventing chronic inflammation. The ratio of bacteria to human cells is approximately 1 : 1 based on the recent estimates, most of them are from the intestines, with an amount of about 10^14^ bacteria [77]. These bacteria form the gut microbiota [78], which plays an important role in human health and disease [79].

ILCs and the gut microbiota communicate with each other in an indirect manner via cytokine signaling, and these signals also combine with signals from intestinal epithelial cells (IECs) and macrophages. The symbiotic microflora can influence the differentiation of ILCs by inducing the expression of intestinal cytokines. Moreover, ILCs react to the gut microbiota by changing their structure, having protective or destructive effects on gut immunity. Among ILCs, the most important with regard to the gut microbiota are ILC3s. Signals that originate from commensal microorganisms affect the maturation of ILCs and the acquisition of the tissue-specific functions by ILCs. Many studies have shown that the microbiota is indispensable for differentiating ILCs and producing IL-22 [50]. IL-22 production by ILC3s also maintains the balance of the microbiota during early colonization resistance against pathogens [80]. Moreover, ILC3s are known to produce IL-22 to protect the body during *Citrobacter rodentium*-induced colitis [50], and IL-22 drives antimicrobial peptide expression and is required for the prevention of severe intestinal pathology and mortality during *C. rodentium*-induced colitis [81]. Interestingly, even in lymphocyte-replete hosts, mice lacking ROR*γ*t^+^ ILCs die from *C. rodentium* infection, although IL-22 can also be produced by Th-17 cells [82].

The gut microbiota can stimulate macrophagocytes to secrete IL-*β*, which can induce ROR*γ*t^+^ ILCs to produce IL-22 [83]. Commensal bacteria directly interact with IECs. Additionally, a germ-free mouse experiment showed that the process of IL-7 secretion by IECs depends on the gut microbiota and that IL-7 is indispensable for promoting cytokine secretion from ILC3s [83]. IECs also secrete IL-25 following stimulation by gut microbiota, and IL-25 decreases the production of IL-22 by ILC3s [83]. IL-22 produced by ILC3s can promote the production of antimicrobial peptides (AMPs) secreted by IECs, thereby limiting symbiotic bacteria, and can regulate the anatomic location of lymphoid symbiotic bacteria [81, 84, 85]. Furthermore, IL-22 derived from ILC3s induces fucosylation in IECs, promoting host protection against enteric pathogens [86] and facilitating the establishment of a healthy intestinal microflora by inhibiting the growth of pathogenic bacteria and conditioned pathogens and preventing damage to the gut tissue [87]. In addition, microbial sensing and production of IL-1*β* by intestinal macrophages drive the secretion of CSF2 by ILC3s, which is needed for macrophage function and the stimulation of oral tolerance [63]. Additionally, the levels of specific *Alcaligenes* IgG in pediatric patients with CD are significantly increased, and the production of IL-22-dependent AMPs inhibits intestinal *Alcaligenes*, indicating that ILC3s play important roles in maintaining intestinal microenvironmental homeostasis [88].

AHR is essential for stimulating innate gut immunity by controlling ROR*γ*t^+^ ILCs [89, 90]. ROR*γ*t^+^ ILCs increased apoptosis in AHR-deficient mice, together with less production of IL-22 and the mice were particularly prone to infection with *C. rodentium* [89, 91]. Consumption of ILCs leads to infection by commensal bacteria and systemic inflammation, and these events can be suppressed by modulation of IL-22 [88]. Based on these results, ROR*γ*t^+^ ILC3s and IL-22 secretion play vital roles in intercommunication among cells [88, 92]. Importantly, ILCs and symbiotic microbes can immediately intercommunicate via Toll-like receptor (TLR) activation. TLR2 expressed on the surface of CD127^+^ LTi-like ILCs can identify bacterial signals, permitting direct sensing of microbial cells [93]. ILCs may also interact with bacterial components through NCRs [94]. For example, NKp46 and NKp44 have been shown to immediately bind to epitopes of *Fusobacterium nucleatum* or *Mycobacterium tuberculosis* [95]. The activity of other ILC subsets can also be affected by microbiota. ROR*γ*t^+^ ILCs are stimulated by epithelial tuft-cell-derived IL-25 [18] in a microbiota-dependent manner [96]. Furthermore, crosstalk between DCs and ILCs would be important in the regulation of intestinal homeostasis, such as confrontation with *H. typhlonius*-driven inflammation, and T-bet, a T-box family transcription factor, plays a crucial role in regulating this interaction [97].

## 4. Activation of ILCs by the ENS

ENS, enteric nervous system, is one of the main divisions of the autonomic nervous system and consists of a mesh-like system of neurons that governs the function of the gastrointestinal tract, it also acts a significant role in the activation of ILCs. The ENS includes the myenteric nerve plexuses and submucosal plexuses and can act alone or in combination with exogenous neurons, storing various nerve programs of gastrointestinal behavior patterns, to regulate almost all functions of the intestine, including exercise, nutrient absorption, immune responses, and blood supply. Moreover, the ENS can manipulate the intestinal tract to release various intestinal hormones, thereby affecting all organs within the body. Besides the brain, the ENS is the most complex nervous system and is sometimes called the “intestinal brain”. The interaction between the ENS and immune system has been reported; however, the relationship between the ENS and ILCs is still unclear. Recent studies have indicated that the ENS plays a role in the activation of ILCs, which then produce cytokines to exert their functions.

A novel process that protects the intestinal lining against inflammation and microbial aggression via interactions between enteric glial cells (EGCs) and ILC3s through neurotrophic factor signals has recently been reported. EGCs are the most abundant and widely distributed cells in the ENS and have been shown to support neuron function and to absorb and regulate certain active substances. Indeed, enteric ILC3 subsets express the neuroregulatory receptor tyrosine kinase (RET), which is activated by glial-derived neurotrophic factor (GDNF) family ligands (GFL) derived from EGCs. Activated RET then sends neural regulation signals to induce IL-22 secretion from ILC3s, thereby mediating intestinal repair. Additionally, neurotrophic factors also directly regulate innate IL-22 downstream of the STAT3 activation. These mechanisms then promote efficient gut homeostasis and defense [22]. Thus, the nervous system acts as a surveillance mechanism for the immune system. When nerve cells receive an alert from the gut, specific instructions are passed on to ILCs to produce effectors that can repair intestinal damage.

In mice, ILC2s, which are essential for immune responses to parasites, have been shown to express a receptor that binds to the neuronal messenger neuromedin U (NMU). Neurons produce large amounts of NMU, and neurons in the intestinal mucosal tissue, as important components of the ENS, rapidly generate NMU following detection of products secreted by helminth, NMU then actively transduces a signal to ILC2s by binding to NMU receptor 1 (NMUR1) on ILC2s. This results in secretion of innate type 2 cytokines to produce a rapid immune response at intestinal mucosal sites [21]. Some other researchers have also found that ILC2s in the intestinal tract of mice colocalize with cholinergic neurons, which produce NMU. In addition to NMUR1, the trigger for this signaling pathway is also dependent on cell-intrinsic expression of G_*α*q_ protein. Furthermore, ILC2s were found to coevolve with the ENS to selectively regulate early sensing and responsiveness to infectious and external stimuli at barrier surfaces [20].

**Figure 2:**
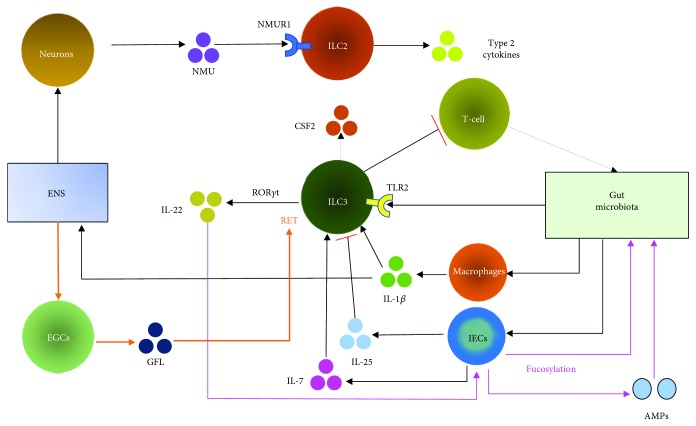
The main relationships between ILCs and the gut microbiota and between ILCs and the ENS in IBD. The gut microbiota can stimulate macrophagocytes to secrete IL-*β*, which can induce ROR*γ*t^+^ ILCs to produce IL-22. This process is also observed for IL-7, which is secreted by intestinal epithelial cells (IECs). IL-25, which is produced by IECs and stimulated by the gut microbiota, decreases the production of IL-22 by ILC3s. IL-22 can also induce fucosylation, which is required for host protection against enteric pathogens, in IECs. Microbial sensing and production of IL-1*β* by intestinal macrophages drive CSF2 secretion by ILC3s. Toll-like receptor 2 (TLR2) expressed on the surface of ILC3s can identify bacterial signals. ILC3 subsets express the neuroregulatory receptor tyrosine kinase (RET), which is activated by glial-derived neurotrophic factor family ligands (GFL) derived from enteric glial cells (EGCs), in response to IL-22 secretion. Neuronal messenger neuromedin U (NMU), produced by neurons, binds to NMUR1 on ILC2s, and ILC2s are activated to secrete innate type 2 cytokines. Activation of ILCs via the ENS or induction of cytokines via the gut microbiota can modulate the development or progression of IBD, as detailed in Figure 1. The gut microbiota is also associated with the ENS. Cytokines regulated by gut microbiota could modulate ENS activity directly or indirectly, e.g., IL-1*β*, which has its receptors on ENS neurons.

Another study suggested that the ENS maintains intestinal health by regulating the constitution of the gut microbiota. The experimental results indicated that the number of proinflammatory bacteria increased significantly in the ENS-dysplastic zebrafish intestine, whereas the number of anti-inflammatory bacteria decreased obviously, resulting in increased incidence of IBD [98]. The main relationships between ILCs and the gut microbiota and between ILCs and the ENS in IBD are shown in Figure 2.

## 5. Conclusions and Future Directions

ILCs have been extensively studied in recent years, and there are many conflicting reports on ILC characteristics and functions. ILCs are relatively rare cells in lymphoid tissue, but participate in a variety of biological functions, particularly in the bowel [99]. Moreover, ILCs play pivotal roles in elimination of infectious pathogens, control of inflammation, and induction of sepsis. In addition to maintaining the homeostasis of the intestinal mucosal barrier in the normal intestinal environment, ILCs also participate in the development and progression of IBD when the intestinal environment changes. Thus, ILCs have dual roles related to anti-inflammatory and proinflammatory responses. Overall, the mechanisms mediating the functions of ILCs are mostly related to the secretion of cytokines and the activities of transcription factors.

The gut microbiota and ENS have important roles in the gut, which are linked through ILCs. Neurons and EGCs (important components of the ENS) can induce ILCs to secret inflammatory cytokines (such as IL-22). Additionally, the gut microbiota interacts with some cells (such as T cells and IECs), which can induce or inhibit the secretion of inflammatory cytokines by ILCs. The release of these cytokines can also alter the gut microbiota. Several reports have also suggested links between the gut microbiota and ENS, potentially involving ILCs [100, 101].

However, there are still many limitations to studies on ILCs. First, the boundaries between the groups of ILCs are still obscure. Expression profiles of cytokines and transcription factors in some ILC subsets are not stable, and mutual transformation may occur among the groups of ILCs [43]. In addition, there is a close relationship between ILCs and Th cell subsets, which function through the same cytokines, thereby making it difficult to clarify the specific intercellular relationships and to study the functions and mechanisms of each ILC subset separately. Existing data indicate that the nervous system sends out specific instructions to the immune system after nerve cells are stimulated by signals from the intestine. Based on such research, the ENS is known as the “second brain” and exhibits autonomic regulation of intestinal motility and secretion. However, further studies are needed to analyze the relationship between the nervous system and the immune system, including basic research studies on the molecular mechanisms. Neurons secrete NMU, which stimulates ILC2s and induces immune responses within a few minutes. This observation may have practical applications in the clinical setting with regard to the immune responses produced after vaccination, which typically require a few weeks. Moreover, most studies on ILCs have been carried out in mice, e.g., the established developmental hierarchy from a CLP and from more restricted precursors of different ILC lineages in mice, such as NKp, ILC1p, ILC2p, and ILC3p. These topics must also be examined in humans. However, although it is now clear that human CLP-like CD34^+^ progenitors from different compartments can give rise to distinct ILC lineages, only NKp and ILC3p have been described in humans. Further in vitro and in vivo studies on human ILCs are needed to elucidate how ILCs operate in the human body. Such research will help us to understand the occurrence and development of IBD and to explore effective treatment strategies to combat related diseases in the future.

## Abbreviations

ILCs: Innate lymphoid cells
IBD: Inflammatory bowel disease
ENS: Enteric nervous system
CLP: Common lymphoid progenitor
IL: Interleukin
Id2/3: Inhibitor of DNA binding 2/3
NK: Natural killer
LTi: Lymphoid tissue inducer
Th: T helper
IFN-*γ*: Interferon-*γ*
TNF-*α*: Tumor necrosis factor-*α*
GATA-3: Gata binding protein 3
RAR: Retinoic acid receptor
ROR*α*: Related orphan receptor-*α*
CSF2: Colony stimulating factor 2
CD: Crohn's disease
UC: Ulcerative colitis
RA: Retinoic acid
cNK: Conventional NK
NCR: Natural cytotoxicity receptor
AHR: Aromatic hydrocarbon receptor
STAT3: Signal transducer and activator of transcription 3
MHCII: MHC class II gene
TGF-*β*: Transforming growth factor
IECs: Intestinal epithelial cells
TLR: Toll-like receptor
EGCs: Enteric glial cells
RET: Receptor tyrosine kinase
GFL: Glial-derived neurotrophic factor family ligands
NMU: Neuronal messenger neuromedin U
NMUR1: NMU receptor 1
G_*α*q_: G-protein *α*-subunit q.
